# Management of dog bites by frontline service providers in primary healthcare facilities in the Greater Accra Region of Ghana, 2014–2015

**DOI:** 10.1186/s40249-018-0398-3

**Published:** 2018-02-28

**Authors:** Ernest Kenu, Vincent Ganu, Charles Lwanga Noora, Richard Adanu, Margaret Lartey

**Affiliations:** 1 Department of Medicine, School of Medicine and Dentistry, College of Health Sciences, University of Ghana, Korle Bu, Accra, Ghana; 2 Department of Epidemiology and Disease Control, School of Public Health, College of Health Sciences, University of Ghana, Legon, Accra Ghana; 3 Department of Medicine and Therapeutics, Korle Bu Teaching Hospital, Accra, Ghana; 4 Sunyani Regional Hospital, Ghana Health Service, Sunyani, Ghana; 5 Department of Population Family and Reproductive Health, School of Public Health, College of Health Sciences, University of Ghana, Legon, Accra Ghana

**Keywords:** Dog bites, Rabies, Frontline service providers, Healthcare facilities, Greater Accra Region, Ghana

## Abstract

**Background:**

Dog bites are common in developing countries including Ghana, with the victims often being children. Although some breeds of dogs have been identified as being more aggressive than others, all dog bites carry a risk of infection. Immediate and initial assessment of the risk for tetanus and rabies infection with appropriate interventions such as wound management and subsequent selection of prophylactic antibiotics are essential in the management of dog bites. This study examined the management of patients with dog bites by frontline service providers at primary healthcare facilities in the Greater Accra Region, Ghana.

**Methods:**

We conducted a cross-sectional study in 66 public health facilities in the Greater Accra Region from July 2014 to April 2015. Up to four frontline service providers were randomly selected to participate from each facility. A structured questionnaire was administered to all consenting participants. Continuous variables were presented as means and standard deviations. The frontline service providers’ knowledge was assessed as a discrete variable and values obtained presented as percentages and proportions. The chi-square test of proportions was used to determine any significant associations between the various categories of the frontline service providers and their knowledge about the management of rabies.

**Results:**

Regarding the frontline service providers’ knowledge about rabies, 57.8% (134/232) were correct in that the rabies virus is the causative agent of rabies, 39.2% (91/232) attributed it to a dog bite, 2.6% (6/232) did not know the cause, and one person (0.4%) attributed it to the herpes virus. Only 15.5% (36/232) knew the incubation period in dogs and the period required to observe for signs of a rabies infection. With respect to the administration of rabies immunoglobulin, 42.2% (98/232) of the frontline service providers did not know how to administer it. Of the facilities visited, 76% (50/66) did not have the rabies vaccines and 44% (102/232) of frontline service providers did not know where to get the rabies vaccines from. Most of the service providers (87.9%; 204/232) had never reported either a dog bite or a suspected case of rabies. Overall, there was gross underreporting of dog bites and suspected rabies cases at public healthcare facilities in the Greater Accra Region of Ghana.

**Conclusions:**

In view of the high morbidity and mortality associated with bites from rabid dogs and the poor knowledge and practices of frontline service providers, there is an urgent need for capacity-building such as training in the management of dog bites and subsequent potential rabies infection.

**Electronic supplementary material:**

The online version of this article (10.1186/s40249-018-0398-3) contains supplementary material, which is available to authorized users.

## Multilingual abstract

Please see Additional file 1 for translations of the abstract into the five official working languages of the United Nations.

## Background

The use of dogs as pets or companions, and for security, hunting, and breeding has contributed to their growing population [1]. The increasing numbers of dogs not owned by individuals and not properly housed has led to an astonishing number of dog bites, which is the main source of rabies [2, 3]. Infected animals may show symptoms such as lethargy, fever, vomiting, and anorexia. These progress to cerebral dysfunction, excessive salivation, abnormal behaviour, aggression, and paralysis, among others [4]. A large proportion of dog bites occur in children and are likely to be from rabid dogs, the main source of the rabies virus infection [1, 3].

Rabies is a fatal zoonotic infection of the central nervous system caused by a lyssavirus. Transmission to humans occurs through contact of infected saliva with open wounds, typically through an animal bite, however, human-to-human transmission can also occur [5, 6]. It is a vaccine-preventable disease and vaccinating dogs is the most cost-effective strategy for preventing rabies in humans [7]. In humans, there are two main forms of the disease: furious rabies and paralytic rabies. In furious rabies, a patient exhibits signs of aerophobia, hydrophobia, hyperactivity, and excited behaviour, dying from a cardio-respiratory arrest a few days later. In paralytic rabies, a patient’s muscles gradually become paralyzed, starting from the site of the bite or scratch, progressing into a coma, and culminating in death [8].

Between 1986 and 2003, Ghana recorded 144 deaths countrywide from rabies from dog bites. Between January 2009 and July 2011, 25 human rabies deaths were recorded [9]. Although Ghana vaccinated 120 933 dogs against rabies in 2011, the total population of dogs in that year was unknown [9]. Meanwhile, the Greater Accra Region alone recorded 2620 dog bites between 2003 and 2008, of which 232 (8.9%) tested positive for rabies. The 2015 study conducted in Ghana by Johnson et al. reported an average of three dogs per household. Unfortunately, these stray dogs were not owned by individuals and were found to be roaming freely, leading to an increase in the number of dog bites, with its accompanying risks including rabies [1].

A person’s risk of infection with rabies virus must be addressed immediately if he/she is bitten by a dog [10]. The wound must be examined to see if there is a laceration or puncture, or if the area is crushed or avulsed. The wound should be washed with soap and water, detergent or povidone-iodine, or any other substance that kills the rabies virus [7]. Administration of potent and effective rabies vaccines and rabies immunoglobulin (RIG) that meets World Health Organization (WHO) standards is required. Treatment with an appropriate antibiotic is important for 3–7 days to tackle potential bacterial infection, and tetanus immunization should be given where indicated. In Ghana, pre-exposure prophylaxis (PrEP) vaccines meeting WHO standards are available and are supplied by the Ministry of Food and Agriculture to the Veterinary Services Directorate. The Ministry of Health only supplies vaccines for post-exposure prophylaxis (PEP) to primary healthcare facilities.

Knowledge and practices of healthcare workers in relation to probable rabies bites do contribute immensely to these bites resulting in poor outcomes in patients despite adequate supplies of vaccines for prevention. This was confirmed by a study done in Karachi, Pakistan, which revealed that among 151 general practitioners, only 19.4% had appropriate knowledge about the first-line treatment for rabies bites, almost all (98%) had no knowledge about types of anti-rabies vaccines, and only 19.2% knew about anti-rabies serum [11].

Most of the observed rabies deaths occurring in the Korle-Bu Teaching Hospital in Ghana from 2010 to 2013 were in individuals referred from primary healthcare settings within the metropolis, in which medical doctors, medical assistants, and nurses work. These patients had sought care immediately after being bitten. Most received the anti-tetanus serum and antibiotics, but unfortunately no anti-rabies treatment was administered to them despite them reporting they were bitten by a dog.

Healthcare workers remain the last port of call through which rabies can be prevented after a suspected bite. Since most affected persons are young and active, adequate treatment will overall help to reduce avoidable deaths and mortality. Although rabies is not a reportable communicable disease in Ghana, it risks becoming a resurgent neglected tropical disease and as such interventions need to be initiated to prevent this resurgence. Considering the near 100% case fatality rate and the fact that preventable vaccines are available, this study sought to examine the knowledge and practices of frontline healthcare workers relating to the management of patients with dog bite wounds at primary healthcare facilities within the public health system in the Greater Accra Region of Ghana.

## Methods

### Study design

A cross-sectional study was carried out at public healthcare facilities in all six administrative districts of the Greater Accra Regional Health Directorate from July 2014 to April 2015.

### Study population and sampling

All 66 public sector healthcare facilities in the Greater Accra Region were included in the study. Frontline health service providers were defined as those looking after casualty wards, emergency rooms, and general outpatient departments. The frontline service providers included met the WHO classification of healthcare service providers. Their job descriptions were matched according to international standards; these were medical doctors, nurses, and medical assistants [12].

A minimum of two and a maximum of four frontline service providers were randomly selected from each facility after they were informed about the study and their consent was sought. Questionnaires were administered after obtaining their written informed consent. Where there were less than four providers in a facility, all were given the chance to be part of the study. In facilities where they were more than four providers, four were selected using simple random sampling from the total number working there.

### Data collection

A structured questionnaire was used, with pre-testing conducted at the Korle-Bu Teaching Hospital. The questionnaire elicited the following information: demographic characteristics of the respondents; knowledge regarding categorization of animal bite wounds, sites, and routes; knowledge regarding the schedule of and guidelines pertaining to PEP; knowledge regarding animal bite wound management; and knowledge regarding RIG administration in routine and special situations. Trained research assistants administered the questionnaires.

### Data handling and analysis

Data were entered, cleaned, coded, and analysed using Stata 13, StataCorp, Texas, USA. Demographic characteristics of the respondents were presented as graphs and tables. Proportions were used to determine adequate knowledge of primary healthcare workers about the management of dog bites, pre-disposing risk factors and clinical presentation of rabies, suspected rabies wound care, and rabies PEP administration. The associations between the various types of healthcare service providers and their levels of knowledge were determined.

Chi-square tests of proportion were conducted to detect any statistical significance between the cadre of service providers and their knowledge about the management of rabies. A simple logistic regression model was used to show the strength of association at 95% confidence intervals (*CI*s). The variables that were significant were entered into a multiple logistic regression model to detect significant determinants.

### Ethical considerations

Ethical approval for the study was obtained from the Ethical Review Committee of the Ghana Health Service and Noguchi Memorial Institute for Medical Research. Furthermore, permission to conduct the study was sought from the Greater Accra Regional Health Directorate and the various public sector health facility superintendents.

Identifiers in terms of healthcare service providers’ and facilities’ names were all removed prior to storage and analysis, and only study IDs were used to guarantee participant and health facility confidentiality.

All members of the study team were required to participate in and have an active certificate in human subject studies and research ethics from the Collaborative Institutional Training Initiative program.

Written informed consent was sought from all participants prior to questionnaire administration. Data were stored in a secure locked cabinet and access was granted to the principal investigator and co-principal investigators only. Data held on computers were encrypted with a password that was made available only on-a-need-to-know basis.

## Results

### Demographic characteristics of the study participants

Two hundred and thirty-two frontline service providers from 66 facilities provided answers to the questionnaires. The mean age of respondents was 33.6 ± 10.1 years (range: 20–65 years). The professional groupings of the respondents were as follows: 75.4% (175/232) were nurses, 13.8% (32/232) were medical assistants, and only 10.8% (25/232) were medical doctors. Twenty-five percent (58/232) of the respondents owned dogs and 75.9% (44/58) of those dogs were vaccinated (see Table 1).

**Table 1:** Demographic characteristics of the study participants

Variable	Frequency (%)
Sex	
Female	185 (79.7)
Male	47 (20.3)
Age	
20–29	116 (50.0)
30–39	66 (28.4)
40–49	22 (9.5)
50 +	28 (12.1)
Profession	
Medical doctor	25 (10.8)
Medical assistant	32 (13.8)
Nurse/midwife	175 (75.4)
Marital status	
Single	102 (44.0)
Married	130 (56.0)
Dog ownership	
Owns a dog	58 (24.6)
Does not own a dog	175 (75.4)
Vaccination status of dog	
Vaccinated	44 (75.9)
Not vaccinated	14 (24.1)

### Frontline service providers’ general knowledge about rabid animals

All frontline service providers interviewed had heard of rabies; 57.8% (134/232) correctly stated that the rabies virus is the causative agent, about 42% (98/232) mentioned other possible causative agents. Some of these possible causative agents mentioned were: 92.9% (91/98) mentioned dog bites, 6.1% (6/98) did not know the cause, and one person (1.0%) said rabies was caused by the herpes virus.

In assessing how long it takes an infected animal to start showing signs and symptoms after a bite, 40.9% (95/232) of the respondents said between 2 and 7 days, 15.5% (36/232) said 10 days, 21.6% (50/232) said 21 days, and the rest did not know. Regarding the period a dog known to have bitten a person without provocation should be observed for, the responses given ranged from 1 day (0.9%; 2/232) to 10 weeks (21.6%; 50/232) (see Table 2).

**Table 2:** Frontline service providers’ knowledge about the management of dog bites

Variable	Frequency (%)
Detain and clinically observe any suspected rabid dog for…	
One day	2 (0.9)
10 days	14 (6.1)
21 days	113 (48.9)
10 weeks	50 (21.6)
Don’t know	5 (2.2)
Rabies PrEP	
Heard	110 (47.6)
Never heard	105 (45.5)
Don’t know	16 (6.9)
Persons requiring PrEP	
Everybody	41 (17.7)
High-risk individuals	60 (26.0)
Nurses/doctors	24 (10.4)
Farmers	16 (6.9)
Don’t know	90 (38.9)
PrEP immunization schedule	
Days 0, 3, 7, and 21 or 28	70 (30.3)
Days 0, 7, and 21 or 28	27 (11.7)
Days 0, 3, and 7	11 (4.8)
Days 1, 7, and 90	8 (3.4)
Don’t know	115 (49.8)
After dog bite, clean and flush wound immediately with water and soap	
Yes	223 (96.1)
No	9 (3.9)
After dog bite, give PEP	
Yes	96 (41.4)
No	136 (58.6)
After dog bite, give tetanus injection	
Yes	228 (98.7)
No	3 (1.3)
Vaccines used for PEP	
Tetanus injection	189 (81.5)
RIG	66 (28.5)
Rabies vaccines	159 (68.5)
Don’t know	11 (4.7)

### Frontline service providers’ knowledge about the management of dog bites

More than half (121/132) of the respondents had neither heard nor knew of PrEP for rabies. Less than 12% (27/232) knew the correct immunization schedule for PrEP. Regarding the immediate management of a dog bite, 96.1% (223/232) of the respondents said the wound/bite site must be immediately washed with soap and water. Only about 40% (96/232) said PEP should be given after dog bite (see Table 2).

### Frontline service providers’ knowledge about suspected rabies wound care

Most of the service providers (87.9%; 204/232) had never reported either a dog bite or a suspected case of rabies.

About 90% (204/232) of the respondents said that suspected rabies wounds have to be washed immediately with water and antiseptic. In assessing the experiences of service providers, 59.1% (137/232) had previously managed dog bites (see Table 3).

**Table 3:** Frontline service providers’ knowledge about suspected rabies wound care

Variable	Frequency (%)
For any rabies wound…	
Cauterization should be done	8 (3.4)
Suturing should be applied	5 (2.2)
Wash it immediately with water and antiseptic	204 (87.9)
Don’t know	15 (6.5)
Suspected rabies wounds are...	
Classified	35 (15.1)
Not classified	197 (84.9)
Number of categories	
Two	20 (30.8)
Three	12 (18.4)
Four	2 (3.1)
Five	2 (3.1)
Don’t know	29 (44.6)
Suspected rabies wound care	
Attended to case	137 (59.1)
Never attended to a case	95 (40.9)

### Frontline service providers’ knowledge of predisposing risk factors and clinical presentation of rabies

About 80% (185/232) of the respondents indicated that barking like a dog was a sign of rabies. Almost all respondents (99.2%; 230/232) said that the mode of transmission of rabies was through a bite from a rabid animal or dog. Regarding risk factors for rabies infection, 84.1% (195/232) of the respondents stated it is a bite from an unprovoked dog and almost all (98.3%; 228/232) said that dogs were the primary reservoirs of the disease (see Table 4).

**Table 4:** Frontline service providers’ knowledge of predisposing risk factors and clinical presentation of rabies

Variable	Frequency (%)
Signs and symptoms of rabies	
Refusal to feed (Y/N)	75 (32.3)
Convulsion (Y/N)	63 (27.2)
Locked jaw (Y/N)	90 (38.8)
Fever (Y/N)	151 (65.1)
Barking like a dog (Y/N)	185 (79.7)
Hydrophobia (Y/N)	160 (68.9)
Others (Y/N)	107 (46.1)
Mode of transmission	
Human bite	1 (0.4)
Snake bite	1 (0.4)
Bite from a rabid animal	230 (99.2)
Risk factors for rabies	
Substance application on umbilical cord	1 (0.4)
Unprovoked dog bite	195 (84.1)
Cut by metal	3 (1.3)
Cut by rusted metal	3 (1.3)
Don’t know	30 (12.9)
Primary rabies reservoir	
Humans	2 (0.9)
Dogs/cats	228 (98.3)
Pigs	0 (0.0)
Camels	0 (0.0)
Cows	2 (0.9)
Don’t know	0 (0.0)

### Frontline service providers’ knowledge about the clinical and public health aspects of rabies PEP

Of the 66 facilities visited, around 76% (50/66) did not have the rabies vaccines and about 44% (102/232) of the service providers did not know where to get the rabies vaccines from. Regarding the duration of the incubation period for rabies, 44.4% (103/232) said it was 3–8 weeks and 26.7% (62/232) did not know, with one abstention. Except for three service providers who did not know how to prevent rabies, 98.7% (229/232) said it was through vaccination. In relation to RIG administration, 42.2% (98/232) of the service providers did not know how it was administered and of those who said they did know, 51.5% (69/134) indicated it was by body surface area, 21.6% (35/134) by body weight, and 0.8% (1/134) by body mass index (BMI) (see Table 5).

**Table 5:** Frontline service providers’ knowledge about the clinical and public health aspects of rabies PEP

Variable	Frequency (%)
Years of practice	
> 1	94 (40.5)
2–5	94 (40.5)
6–9	19 (8.2)
≥ 10	25 (10.8)
Availability of rabies vaccines at facility based on providers’ responses	
Available	54 (23.3)
Not available	178 (76.7)
Knowledge of where to get vaccine	
Yes	129 (55.8)
No	102 (44.2)
Incubation period of rabies	
One day	4 (1.7)
6 days	23 (9.9)
One week	39 (16.8)
3–8 weeks	103 (44.4)
Don’t know	63 (27.2)
Rabies prevention	
Vaccination	229 (98.7)
Prayers	0 (0.0)
Kill all animals	0 (0.0)
Other	0 (0.0)
Don’t know	3 (1.3)
Dose of RIG determined by…	
BMI	1 (0.8)
Weight	29 (21.6)
Body surface area	69 (51.5)
Don’t know	35 (26.1)
Site for RIG administration	
Infiltrated around the wound	48 (20.7)
Intravenous	7 (3.0)
All dosed into deltoid muscle	108 (46.6)
Don’t know	69 (29.7)
Site for rabies vaccine	
Deltoid	134 (57.8)
Abdomen	25 (10.8)
Buttocks	22 (9.5)
Don’t know	51 (21.9)
Route of rabies vaccination	
Intramuscular	115 (49.6)
Intradermal	43 (18.5)
Intramuscular/intradermal	32 (13.8)
Intravenous	1 (0.4)
Don’t know	41 (17.7)

### Association between knowledge about the management of dog bites and the cadre of frontline service providers

In the bivariate analysis, knowledge of rabies PrEP (*P* <  0.01), whether suspected rabies bites are classified (*P* <  0.001), and the number of rabies wound categories (*P* <  0.01) were variables significantly associated with the professional category of service providers. However, in the multiple logistic regression analysis, none of these factors were significantly associated with provider category (see Table 6).

**Table 6:** Association between cadre of frontline workers and knowledge on management of dog bites, Greater Accra region

Variable	Medical doctor (%)	Other Health Staff (%)	cOR (95% CI)	P value	aOR (95% CI)	P value
Detain and clinically observe any suspect for						
21 days	12 (48.0)	100 (48.8)	1.0		1.0	
Don’t know	13 (52.0)	105 (51.2)	0.9 (0.4-2.2)	0.94	0.6 (0.2-1.8)	0.40
Rabies Pre-exposure prophylaxis (RPP)						
Ever heard	6 (24.0)	114 (55.6)	1.0		1.0	
Never heard	19 (76.0)	91 (44.4)	**0.3 (0.1-0.6)**	**<0.01**	**0.2 (0.1-0.7)**	**0.01**
RPP immunization is scheduled						
Days 0, 7 and 21 or 28	7 (28.0)	63 (30.7)	1.0		1.0	
Don’t know	18 (72.0)	142 (69.3)	0.9 (0.3-2.2)	0.78	0.4 (0.1-1.3)	0.69
After dog bite, Clean and flush wound immediately with soap						
Yes	6 (24.0)	89 (43.4)	1.0		1.0	
No	19 (76.0)	116 (56.6)	0.4 (0.2-1.1)	0.06	0.4 (0.1-1.3)	0.13
For any rabies wound						
Wash immediately with water and antiseptic	24 (96.0)	180 (87.4)	1.0		1.0	
Cauterization and Suturing should be done	1 (4.0)	26 (12.6)	3.5 (0.4-26.7)	0.21	3.0 (0.1-1.7)	0.33
Suspected rabies wounds are						
Classified	15 (60.0)	181 (87.9)	1.0		1.0	
Not classified	10 (40.0)	25 (12.1)	**0.2 (0.1-0.5)**	**<0.001**	0.4 (0.1-1.7)	0.23
Number of categories						
Three	4 (16.0)	8 (3.9)	1.0		1.0	
Don’t know	21 (84.0)	27 (96.1)	**4.7 (1.3-16.9)**	**<0.001**	2.1 (0.3-15.5)	0.45
Rabid wound care						
Ever attended to case	7 (28.0)	83 (40.3)	1.0		1.0	
Never attended to a case	18 (72.0)	123 (59.7)	0.6 (0.2-1.4)	0.23	0.9 (0.3-2.9)	0.90

Bolden figures: significant at *P* < 0.05

*cOR *crude odds ratio, *aOR *adjusted odds ratio

Similarly, other healthcare service providers were less likely to infiltrate around the wound with RIG compared to medical doctors (a*OR* = 0.3, 95% *CI*: 0.1–0.6), but were more likely to administer the vaccine around the deltoid (a*OR* = 3.1, 95% *CI*: 1.2–8.1) (see Table 7).

**Table 7:** Association between cadre of frontline service providers and knowledge about the clinical presentation and risk factors for rabies, Greater Accra Region

Variable	Medical doctors (%)	Other Health Staff (%)	cOR (95% CI)	P value	aOR (95% CI)	P Value
Incubation period of rabies						
3-8 weeks	13 (52.0)	89 (43.4)	1.0		1.0	
Don’t know	12 (48.0)	116 (56.6)	1.4 (0.6-3.2)	0.42	0.7 (0.2-1.9)	0.48
Rabies prevention						
Vaccination	25 (11.0)	203 (99.0)	1.0		1.0	
Don’t Know	0 (0.0)	2 (1.0)	-	0.62	-	
Rabies immunoglobulins are given by						
Weight	8 (32.0)	62 (30.1)	1.0		1.0	
BMI/Body surface area	17 (68.0)	144 (69.9)	1.1 (0.4-2.7)	0.84	0.8 (0.2-2.3)	0.62
Site for rabies immunoglobulin administration						
Infiltrated around the wound	11 (44.0)	37 (18.0)	1.0		10	
All dosed into deltoid	14 (56.0)	169 (82.0)	**3.6 (1.5-8.5)**	**<0.01**	3.1 (0.9-9.8)	0.05
Site for rabies vaccine						
Deltoid	10 (40.0)	124 (60.2)	1.0		1.0	
Abdomen/ Buttocks	15 (60.0)	82 (39.8)	0.4 (0.2-1.1)	0.05	**0.2 (0.1-0.5)**	**<0.01**
Route of rabies vaccination						
Intramuscular/intradermal	17 (68.0)	130 (63.1)	1.0		1.0	
Intravenous	8 (32.0)	76 (36.9)	1.2 (0.5-3.1)	0.63	1.4 (0.4-4.2)	0.58
Mode of transmission						
Bite from rabid animal	24 (96.0)	205 (99.5)	1.0		1.0	
Human bite	1 (4.0)	1 (0.5)	0.1 (0.01-1.9)	0.63	0.1 (0.01-2.4)	0.15
Risk factors for rabies						
Unprovoked dog bite	24 (96.0)	205 (99.5)	1.0		1.0	
Cut by metal/other	1 (4.0)	1 (0.5)	0.1 (0.01-1.9)	0.63	3.6 (0.4-32.3)	0.25
Primary rabies reservoir						
Dogs/cats Y/N	24 (96.0)	171 (82.5)	1.0		1.0	
Other	1 (4.0)	36 (17.5)	5.1 (0.7-38.8)	0.08	1.3 (0.3-5.6)	0.71

Bolden figures: significant at *P* < 0.05

*cOR *crude odds ratio, *aOR* adjusted odds ratio

## Discussion

This study sought to examine the knowledge of frontline service providers about the management of patients with dog bite wounds at public primary healthcare facilities in the Greater Accra Region, Ghana.

The study revealed that more than 70% of emergency care providers at primary healthcare facilities who were most likely to attend to cases of dog bites or rabies were nurses, whilst only about 11% were medical doctors. The average duration of practice of the respondents was 5 years, with around 10% having practiced for more than 10 years. All respondents had heard of rabies.

Generally, less than 50% of the respondents knew how to handle suspected rabid animals and knew about the signs the animals were likely to show if they were infected. Nearly 58% of frontline service providers correctly identified the rabies virus as the causative agent of rabies. This is consistent with earlier findings of a study conducted on the knowledge about rabies among interns at the Geetanjali Medical College in Rajasthan, India [13]. However, the results of this study contradict another study in western India, in which less than 20% of healthcare service providers were found to know the cause of rabies [14].

This study found that more than half of the service providers did not know the rabies incubation period in dogs and subsequently how long to detain and observe a suspected rabid dog. Remarkably, the majority of healthcare providers had only worked at their respective facility for few years and this may have contributed to the low levels of knowledge about dog bite management among them. Arguably, the lack of continuous professional development for these providers could be a major contributor to this. Continuous development activities such as training and retraining of healthcare service providers in the management of dog bites and rabies prophylaxis are needed to fill their knowledge gap [15]. This can be achieved if the Africa Rabies Expert Bureau (AfroREB) makes rabies a reportable disease, establishes rabies control programs, and if a close collaboration between human health and veterinary health is adhered to [16].

Regarding the immediate management of dog bite wounds, 95.6% of the respondents said wound/bite sites must be immediately washed with soap/antiseptic and water. This is in line with the WHO recommendations for rabies wound management [17]. This proportion was higher than others found in similar studies done in India in 2012 and 2014 i.e. 89% and 19% respectively. [13, 15]. The AfroREB found that many people did not know what to do in the event of a dog bite [16]. Our study found, however, that a high percentage of providers wash the bite wound with soap and water as that seemed to be the most plausible alternative. Washing a dog bite wound with soap and water is said to reduce the viral load and mortality by as much as 50% [18, 19].

Only 40% of service providers said they would give PEP to anyone with a dog bite. This is an indication that awareness of the importance of PEP among healthcare service providers in the region is on the low side. This is worrying since PEP is known to save lives if given in a timely manner [20, 21].

Less than 40% of service providers did not know who requires rabies PrEP, which is similar to findings from a study conducted on knowledge and practices in relation to animal bite management and rabies prophylaxis among doctors in Delhi, India [15].

This study also showed that more than 45% of primary healthcare service providers in the Greater Accra Region had never heard of rabies PrEP. This revealed a lack of knowledge in the management of dog bites and once again these findings were similar to that of an Indian study on dog bite management conducted among doctors [15]. Rabies PrEP, if given to the right individuals, can protect them by averting rabies [22]. Our findings were consistent with findings from another study, which found that more than 52% of service providers did not know about PrEP or who was eligible for it. From our study, a small percentage (6.9%) of the providers believed that farmers were the ones who would most likely require PrEP, while a significant proportion (17.7%) suggested that everyone is eligible for PrEP and 26.0% indicate only high risk individuals should be given PrEP. Effective rabies control requires massive campaigns on vaccination of dogs and PrEP vaccinations for at-risk populations. Controlling the disease in the dog population through vaccination has proven to be cost-effective and efficient.

Rabies is mainly transmitted via a bite from a rabid infected animal and most, if not all, healthcare providers are expected to know the mode of transmission. Most of the providers surveyed did know the cause of rabies as in a study conducted by Mishra in 2014 [13]. Contrary to these findings, less than 50% of the respondents in our study correctly indicated that the incubation period for rabies is 3–8 weeks in humans (and could be longer). This was comparable to the 51.7% result obtained in a study conducted in Pakistan [10]. Our finding indicates some of the gaps in the knowledge of rabies among frontline healthcare service providers in Ghana.

Only 15.1% of the service providers surveyed knew the right way to categorize suspected rabies wounds. This is a problem as the category of wound determines the appropriate treatment [17]. In contrast, a study conducted in North India found that 50% of service providers had knowledge about the correct categorization of animal bite wounds [23].

Rabies case fatality is nearly 100%, and the disease can only be prevented via vaccination of both animals and humans. With respect to the administration of RIG, more than 40% of the frontline service providers surveyed in this study did not know how the vaccine was administered or the site of administration. This is similar to what was observed among healthcare service providers in Pakistan [12]. In this era of many emerging and re-emerging zoonotic diseases, the knowledge gaps among healthcare service providers in terms of rabies management in particular is of concern. It is important to know the status of other zoonotic diseases. This gap also signals that a weak collaboration between primary healthcare providers and veterinary departments exists, a sign of likely challenges for the country when faced with a zoonotic disease outbreak [24].

Our study found that over 50% of service providers were aware of the anti-RIG dose to be administered. This is similar to results obtained by Shashikantha, who showed that 52% of service providers were aware of the anti-RIG dose [23].

This study also found that 76% of the public sector healthcare facilities visited did not have rabies vaccines in stock and 44% of the service providers did not know where to procure the rabies vaccines. This finding confirmed a study conducted in India, which found the use of rabies vaccines to be very low [14]. Unavailability of treatment, and delayed or incomplete treatment are factors that are likely to increase morbidity and lead to fatalities [25].

In terms of reporting either dog bites or suspected cases of rabies, around 88% of service providers had never done this. There were no records of dog bite cases providers have attended to. This makes it difficult to ascertain whether they have actually managed cases, thereby increasing the likelihood of complications of the dog bite including rabies. Dodet and colleagues reported that most bitten individuals in Africa do not get full immunization due to a lack of information, limited resources, and poor accessibility to rabies prevention centres [16]. All these contribute to the incidence of rabies.

Having controlled for all confounding variables, we found that medical doctors were more likely to infiltrate around the wound compared to other healthcare service providers. This could be because medical doctors undergo a longer and more detailed training compared to the basic training other healthcare service providers get [12]. Considering that other healthcare providers comprise almost 90% of the frontline service providers in the Greater Accra Region, it is very prudent that measures be put in place by the Regional Health Directorate to improve the frontline health staff’s knowledge in order to better address this important and urgent issue.

### Study limitations

This study surveyed healthcare service providers, with the focus on health care. The veterinary aspect was not studied. In line with the One Health concept, future studies would need to look at both aspects to obtain a holistic picture and thus establish appropriate interventions.

## Conclusions

In this study, although most frontline service providers at hospital emergency points in the Greater Accra Region knew the cause of rabies, only about 40% knew about the first-line treatment. This study revealed extensive gaps in the knowledge pertaining to and inadequate management of dog bites among frontline healthcare service providers. The observed inadequacies in the management of dog bites might have led to some vaccine-preventable deaths. Lack of vaccines in the health facilities was also a main challenge identified in the study.

To adequately address the observed gaps in the management of dog bites and prevention of rabies, the following must be implemented: A concerted effort by health and veterinary services must be initiated to improve on the knowledge and practices of frontline workers in the management of animal bites and the prevention of rabies. This is important because health is the second line of prevention of rabies in humans if the first line of prevention (among animals) fails. We recommend that more efforts are made by the health service to educate service providers, and stock facilities with vaccines and ensure they are used appropriately to prevent rabies in Ghana.

## Additional file

Additional file 1: Multilingual abstract in the five official working languages of the United Nations. (PDF 474 kb)

## Abbreviations

AfroREB: Africa Rabies Experts Bureau
a*OR*: Adjusted odds ratio
BMI: Body mass index
*CI*: Confidence interval
c*OR*: Crude odds ratio
PEP: Post-exposure prophylaxis
PrEP: Pre-exposure prophylaxis
RIG: Rabies immunoglobulin
WHO: World Health Organization
