# Supplementary material for: Management of dog bites by frontline service providers in primary healthcare facilities in the Greater Accra Region of Ghana, 2014–2015
Source: Infect Dis Poverty. 2018 Feb 28;7:18. doi: 10.1186/s40249-018-0398-3 (PMC5836462; doi:10.1186/s40249-018-0398-3)
Supplement: Supplementary file 1 — Multilingual abstract in the five official working languages of the United Nations. (PDF 474 kb) [file 40249_2018_398_MOESM1_ESM.pdf]

Translation of the abstract into the five official working languages of the United Nations

إدارة عضات الكلاب التي يقوم بها مقدمو خدمات الخط الأول في مرافق الرعاية الصحية في منطقة أكرا الكبرى في غانا، 2014- 2015

إرنست كينو،و فنست غانو، وتشارلز لوانجا نورا، وريتشارد ادانو، ومارجريت لارتي

الملخص

نبذة: تشيع عضات الكلاب في الدول النامية بما فيها غانا، وغالبا ما يكون ضحاياها الأطفال ورغم أن بعض فصائل الكلاب تعرف بأنها أكثر عدائية من الأخرى، إلا أن كافة عضات الكلاب تحمل خطر الإصابة بالمرض .

إن التشخيص الفوري والأولي لخطر الإصابة بالكزاز(التيتانوس) و داء الكَلَب المصحوب بالتدخل الملائم كإدارة الجروح وما يصحبها من اختيار مضاد حيوي وقائي هو أمر رئيسي في إدارة عضات الكلاب.

تفحصت هذه الدراسة إدارة مقدمي خدمة الصف الأول للمرضى المصابين بعضات كلاب في المرافق الصحية الأساسية في منطقة أكرا الكبرى في غانا.

الطرق :أجرينا دراسة مستعرضة في 66 منشأة صحية عامة في منطقة أكرا الكبرى من تموز 2014 إلى نيسان2015.

أختير ما لا يقل عن أربعة من مقدمي خدمة الصف الأول من كل مرفق صحي بشكل عشوائي للمشاركة، وأجري استبيان منظم لجميع المشاركين الذين أبدوا موافقتهم.

قُدمت المتغيرات المستمرة كوسائل وانحرافات معيارية.

قُيمت معرفة مقدمي خدمة الصف الأول على أساس المتغير المنفصل، وقُدمت القيم المحصلة كنسب موية ونسب عادية.

استخدم نموذج مربع تشاي لاختبار النسب من أجل تحديد أية ارتباطات مهمة بين الفئات المتنوعة لمقدمي خدمة الصف الأول وبين اطلاعهم على إدارة داء الكلب.

النتائج : وفيما يتعلق بمعرفة مقدمي خدمات الخطوط الأمامية لداء الكلب كان 57.8٪ (134/232) من المقدمين محقا في أن فيروس داء الكلب هو العامل المسبب لداء الكلب، وعزا 39.2٪ (91/232)ذلك إلى عضة الكلب، ولم يعرف 2.6٪ (232 / 6) السبب، وعزا شخص واحد 0.4٪ ذلك إلى فيروس الحلأ (الهربس).

عرف فقط 15.5٪ (36/232) فترة الحضانة في الكلاب، والفترة اللازمة لمراقبة علامات الإصابة بداء الكلب.

وفيما يتعلق بإدارة الغلوبولين المناعي لداء الكلب، فإن 42.2٪ (98/232) من مقدمي خدمات الخطوط الأمامية لا يعرفون كيفية إدارته.

من المرافق التي تمت زيارتها، تبين أنه 76٪ (50/66) لم يكن لديهم لقاحات داء الكلب، و 44٪ (102/232) من مقدمي الخدمات في الخطوط الأمامية لم يعرفوا من أين يحصلون على لقاحات داء الكلب .

ومعظم مقدمي الخدمات 87.9٪؛ (204/232) لم يبلغوا قط عن عضة كلب أو حالة مشتبه فيها لداء الكلب .

وبشكل عام، كان هناك نقص في الإبلاغ عن لدغات الكلب وحالات داء الكلب المشتبه فيها في مرافق الرعاية الصحية العامة في منطقة أكرا الكبرى في غانا.

الاستنتاجات

وبالنظر إلى ارتفاع معدلات الاعتلال والوفيات المرتبطة بعضات الكلاب المسعورة، وضعف اطلاع وممارسات مقدمي الخدمات في الخطوط الأمامية، فإن هناك حاجة ماسة إلى بناء القدرات مثل التدريب على إدارة عضات الكلاب وما يصاحب ذلك من إصابة بداء الكلب .

Translated from English version into Arabic by Houtary, through

TRANSLATORS
WITHOUT BORDERS

加纳大阿克拉地区的初级保健机构一线服务提供者的犬咬伤管理

Ernest Kenu, Vincent Ganu, Charles Lwanga Noora, Richard Adanu, Margaret Lartey

摘要

背景：犬咬伤常见于包括加纳在内的发展中国家，受害者往往是儿童。虽然有些品种的犬被认为比其他品种更具攻击性，但所有犬咬伤都有感染的风险。通过伤口处理和选用预防性抗生素等适当的干预手段，对破伤风和狂犬病感染风险进行即时和初步评估在犬咬伤管理中至关重要。本研究对加纳大阿克拉地区基层医疗机构一线服务者对犬咬伤患者的管理进行调查。

方法： 2014 年 7 月至 2015 年 4 月，在大阿克拉地区的 66 家公共医疗机构进行横断面研究。从每个设施随机选择 4 名一线服务者，对同意参与者进行结构化问卷调查。连续变量用均值和标准差表示。一线服务者的知识被评估为离散变量，所获得的值以百分比和比例表示。使用卡方检验来确定各类一线服务者与狂犬病管理知识之间的关联。

结果：关于一线服务者对狂犬病的了解，57.8%（134/232）的人能正确认识狂犬病病毒是狂犬病的致病因子，39.2%（91/232）归因于犬咬伤，2.6%（6/232）不知道病因，1 人（0.4%）归因于疱疹病毒；只有 15.5%（36/232）的人知道狂犬病在犬的潜伏期和观察到狂犬病病毒感染迹象所需的时间。关于狂犬病免疫球蛋白的管理，42.2%（98/232）的人不知道如何管理。在所调查的机构中，76%（50/66）的机构没有狂犬病疫苗，44%（102/232）的一线服务者不知道从哪里获得狂犬病疫苗。大部分一线服务者（87.9%，204/232）从未报告过犬咬伤或狂犬病疑似病例。总而言之，加纳大阿克拉地区的公共医疗机构存在严重漏报的犬咬伤和狂犬病疑似病例。

结论：鉴于高发病率和死亡率与狂犬病病犬咬伤以及一线服务者的知识缺乏和做法有关，迫切需要进行犬咬伤和及其潜在的狂犬病病毒感染管理能力建设的培训。

Translated from English version into Chinese by Qin Chen

CDC CHINESE CENTER FOR DISEASE CONTROL AND PREVENTION
NATIONAL INSTITUTE OF PARASITIC DISEASES

Gestion des morsures de chiens par les prestataires de services de première ligne dans les établissements de soins de santé primaires de la région du Grand Accra au Ghana, 2014 - 2015

Ernest Kenu, Vincent Ganu, Charles Lwanga Noora, Richard Adanu, Margaret Lartey

Contexte : Les morsures de chiens sont courantes dans les pays en développement, y compris au Ghana, les victimes étant souvent des enfants. Bien que certaines races de chiens aient été identifiées comme étant plus agressives que d'autres, toutes les morsures de chien comportent un risque d'infection. L'évaluation immédiate et initiale du risque d'infection par le tétanos et la rage avec des interventions appropriées telles que la prise en charge des plaies et la sélection subséquente d'antibiotiques prophylactiques sont essentielles dans la prise en charge des morsures de chien. Cette étude a examiné la prise en charge des patients ayant des morsures de chien par des prestataires de services de première ligne dans des établissements de soins de santé primaires dans la région du

Grand Accra, au Ghana.
Méthodes : Nous avons mené une étude transversale dans 66 établissements de santé publique dans la région du Grand Accra de juillet 2014 à avril 2015. Jusqu'à quatre fournisseurs de services de première ligne ont été sélectionnés au hasard pour participer à chaque installation. Un questionnaire structuré a été administré à tous les participants consentants. Les variables continues ont été présentées en tant que moyennes et écarts-types. Les connaissances des fournisseurs de services de première ligne ont été évaluées en tant que variable discrète et les valeurs obtenues présentées en pourcentages et en proportions. Le test du chi carré des proportions a été utilisé pour déterminer les associations significatives entre les différentes catégories de fournisseurs de services de première ligne et leurs connaissances sur la prise en charge de la rage.
Résultats : Concernant les connaissances des prestataires de services de première ligne sur la rage, 57,8% (134/232) ont raison : le virus de la rage est l'agent responsable de la rage, 39,2% (91/232) l'attribuent à une morsure de chien, 2,6% (6/232) ne connaissait pas la cause, et une personne (0,4%) l'a attribué au virus de l'herpès. Seulement 15,5% (36/232) connaissaient la période d'incubation chez les chiens et la période nécessaire pour observer les signes d'une infection rabique. En ce qui concerne l'administration de l'immunoglobuline antirabique, 42,2% (98/232) des fournisseurs de services de première ligne ne savaient pas comment l'administrer. Parmi les établissements visités, 76% (50/66) ne disposaient pas des vaccins antirabiques et 44% (102/232) des fournisseurs de services de première ligne ne savaient pas où se procurer les vaccins antirabiques. La plupart des fournisseurs de services (87,9%, 204/232) n'avaient jamais signalé de morsure de chien ou de cas suspect de rage. Dans l'ensemble, il y a eu une sous-déclaration importante des morsures de chien et des cas présumés de rage dans les établissements de santé publics de la région du Grand Accra au Ghana.
Conclusions : Compte tenu de la morbidité et de la mortalité élevées associées aux morsures de chiens enragés et des connaissances et pratiques médiocres des prestataires de services de première ligne, il est urgent de renforcer les capacités telles que la formation à la prise en charge des morsures de chien.

Translated from English version into French by matoudiallo, through

TRANSLATORS
WITHOUT BORDERS

Лечение укусов собак медработниками "первой линии" первичной медицинской помощи в городе Аккра, Гана в 2014 – 2015 г.г.

Эрнест Кену, Винсент Гану, Чарльз Лванга Нур, Ричард Адану, Маргарет Ларти

Аннотация

Справка: Укусы собак распространены в развивающихся странах таких, как Гана; причем жертвами часто становятся дети. Хотя некоторые породы собак более агрессивны, чем другие, все укусы собак несут риск инфицирования. Экстренная первоначальная оценка риска

заражения столбняком и бешенством с помощью соответствующих мероприятий, таких как лечение ран и последующий выбор профилактических антибиотиков, имеют большое значение при лечении укусов собак. В этом исследовании рассматривается вопрос о лечении пациентов с укусами собак медработниками "первой линии" в первичных медицинских учреждениях в городе Аккра, Гана.

Методы: Мы провели исследование методом поперечных срезов в 66 государственных медицинских учреждениях Аккры с июля 2014 по апрель 2015. От одного до четырех медработников "первой линии" были выбраны случайным образом от каждого медучреждения. Все участники согласились со структурированным вопросником. Непрерывные переменные были представлены как средства и как стандартные отклонения. Знания медработников "первой линии" оценивались как дискретные переменные и полученные значения представлены в виде процентных долей и пропорций. Для определения любых значительных ассоциаций между различными категориями медработников "первой линии" и их знаний о лечении бешенства был использован тест пропорций хи-квадрат.

Результаты: Что касается знаний о бешенстве медработников "первой линии", то 57,8% (134/232) правильны в том, что вирус бешенства является возбудителем бешенства, 39,2% (91/232) приписывают его к укусу собак, 2,6% (6/232) не знают причины, и один человек (0,4%) отнес его к вирусу герпеса. Только 15,5% (36/232) знают инкубационный период у собак и период, необходимый для наблюдения за признаками инфекции бешенства. Что касается введения иммуноглобулина бешенства, 42,2% (98/232) медработников "первой линии" не знали, как его применять. Из посещенных объектов, 76% (50/66) не имеют вакцины против бешенства и 44% (102/232) медработников “первой линии” не знают, где получить вакцину от бешенства. Большинство медработников "первой линии" (87,9%; 204/232) никогда не сообщали об укусах собак, или о предполагаемом случае бешенства. В целом, обнаружено общее недоинформирование об укусах собак и потенциальных случаях бешенства в государственных медицинских учреждениях в г. Аккра, Гана.

Выводы: В связи с высокой заболеваемостью и смертностью из-за укусов бешеных собак, и плохих знаний и практики медработников "первой линии" существует настоятельная необходимость в наращивании потенциала, например в подготовке кадров по лечению укусов собак и последующей потенциальной инфекции бешенства.

Translated from English version into Russian by TatianaKary, through

Gestión de las mordeduras de perros por parte de los proveedores de servicios en primera línea en los centros de salud de la región de Gran Acra en Ghana, 2014 - 2015

Ernest Kenu, Vincent Ganu, Charles Lwanga Noora, Richard Adanu, Margaret Lartey

Resumen

Contexto: Las mordeduras de perro son comunes en los países en desarrollo, también en Ghana, y sus víctimas son a menudo niños. Aunque se hayan identificado algunas razas de perro como más agresivas que otras, todas las mordeduras de perro conllevan riesgo de infección. Para una correcta gestión de las mordeduras de perro, es imprescindible una evaluación inicial e inmediata del riesgo de tétanos y rabia utilizando las operaciones adecuadas en lo que se refiere a gestión de heridas y la posterior selección de antibióticos profilácticos. Este estudio examina la gestión de las mordeduras de perros por parte de los proveedores de servicios en primera línea en los centros de salud de la región de Gran Acra en Ghana.

Métodos: Hemos realizado un estudio transversal en 66 centros públicos de salud en la región de Gran Acra desde julio de 2014 hasta abril de 2015. Se seleccionaron al azar hasta cuatro proveedores de servicios en primera línea en cada uno de los centros. Se proporcionó un cuestionario estructurado a todos los participantes que dieron su consentimiento. Se presentaron variables continuas tipo promedios y desviaciones estándar. El conocimiento de los proveedores de servicios en primera línea se evaluó en forma de variable discreta y los valores obtenidos se presentaron en forma de porcentajes y proporciones. Se utilizó la prueba de ji-cuadrado de proporciones para determinar cualquier relación significativa entre las diferentes categorías de proveedores de servicios en primera línea y su conocimiento en relación a la gestión de la infección rábica.

Resultados: En relación al conocimiento de los proveedores de servicios en primera línea sobre la rabia, el 57,8% (134/232) respondió correctamente que el virus de la rabia es el agente causante de la enfermedad de la rabia, el 39,2% (91/232) lo atribuyó a la mordedura de perro, el 2,6% respondió no conocer la causa y una persona (0,4%) lo atribuyó al virus del herpes. Solo el 15,5% (36/232) tenía conocimiento del período de incubación en perros y del tiempo necesario para observar síntomas de infección rábica. Respecto a la administración de inmunoglobulina de la rabia, el 42,2% (98/232) de los proveedores de servicios en primera línea no sabía cómo administrarla. De los centros visitados, el 76% (50/66) no disponía de vacunas contra la rabia y el 44% (102/232) no sabía dónde se podían encontrar. La mayoría de los proveedores de servicios (87,9%, 204/232) nunca había comunicado una mordedura de perro o un posible caso de rabia. En general, se encontró una importante falta de comunicación de mordeduras de perros y posibles casos de rabia en los centros públicos de salud de la región de Gran Acra en Ghana.

Conclusiones: A la vista de la alta morbilidad y mortalidad asociadas con las mordeduras de perros rabiosos y el pobre conocimiento y deficientes prácticas de los proveedores de servicios en primera línea, resulta muy urgente realizar programas de capacitación como la formación en la gestión de mordeduras de perros y sobre el riesgo de infección rábica que conllevan.

Translated from English version into Spanish by Joan Banach, through

TRANSLATORS
WITHOUT BORDERS
